# Supplementary material for: Spatial variation of the rain–snow temperature threshold across the Northern Hemisphere
Source: Nat Commun. 2018 Mar 20;9:1148. doi: 10.1038/s41467-018-03629-7 (PMC5861046; doi:10.1038/s41467-018-03629-7)
Supplement: Supplementary file 1 — Supplementary Information(DOCX 8229 kb) [file 41467_2018_3629_MOESM1_ESM.docx]

**Spatial variation of the rain-snow temperature threshold across the Northern Hemisphere**

Supplemental Material

Jennings et al.

**Supplementary Figure 1.** Observed snow frequency by air temperature (T_s_), wet bulb temperature (T_w_), and dew point temperature (T_d_). T_w_ was calculated via Stull^5^ and T_d_ was measured as part of the observational dataset. The 50% rain-snow temperature threshold was greatest for T_s_ (1.0°C), while both T_w_ (0.3°C) and T_d_ (-0.3°C) were lower.

**Supplementary Figure 2.** Simulated snowfall frequency difference between the trivariate (T_s_, RH, P_s_) and bivariate (T_s_, RH) binary logistic phase regression models. Areas shaded in blue indicate the trivariate model simulated a higher snowfall frequency, while red shading corresponds to a lower simulated snowfall frequency from the trivariate model.

**Supplementary Table 1.** The 90% (*T_90_*), 50% (*T_50_*), and 10% (*T_10_*) rain-snow air temperature thresholds, and the temperature range between *T_90_* and *T_10_* for each RH and P_s_ bin. Each threshold is associated with the percentage of precipitation comprised of snowfall (i.e., at *T_90_*, 90% of observed precipitation falls as snow). Note: No *T_90_* and range value could be calculated for the lowest RH bin because there was no temperature in the observed data from -8°C to 8°C at which snow frequency met or exceeded 90%. This was likely due to the small sample size for this RH bin.

|  | **Bin** | **T_90_ (°C)** | **T_50_ (°C)** | **T_10_ (°C)** | **Range (°C)** |
| --- | --- | --- | --- | --- | --- |
| RH (%) | 40 < RH <= 50 | NA | 4.5 | 6.7 | NA |
|  | 50 < RH <= 60 | 1.0 | 3.7 | 5.6 | 4.6 |
|  | 60 < RH <= 70 | 1.1 | 2.8 | 4.2 | 3.1 |
|  | 70 < RH <= 80 | 0.7 | 2.2 | 3.4 | 2.7 |
|  | 80 < RH <= 90 | 0.0 | 1.4 | 2.6 | 2.6 |
|  | 90 < RH <= 100 | -0.7 | 0.7 | 1.9 | 2.6 |
| P_s_ (kPa) | 60 < P_s_ <= 70 | 0.3 | 1.9 | 3.6 | 3.3 |
|  | 70 < P_s_ <= 80 | 0.2 | 1.7 | 3.3 | 3.1 |
|  | 80 < P_s_ <= 90 | -0.4 | 1.3 | 2.8 | 3.2 |
|  | 90 < P_s_ <= 105 | -0.7 | 0.9 | 2.4 | 3.1 |
|  |  |  |  |  |  |

**Supplementary Table 2.** Optimized coefficients for the univariate (T_s_), bivariate (T_s_-RH), and trivariate (T_s_-RH-P_s_) binary logistic precipitation phase prediction models with the coefficient standard deviations in parenthesis. Coefficient symbols correspond to those presented in the Methods sections in equations (2), (3), and (4).

| **Model** | ***α*** | ***β* (T_s_)** | ***γ* (RH)** | ***λ* (P_s_)** |
| --- | --- | --- | --- | --- |
| Univariate | -1.54 (0.06) | 1.24 (0.04) | NA | NA |
| Bivariate | -10.04 (0.68) | 1.41 (0.05) | 0.09 (0.007) | NA |
| Trivariate | -12.80 (1.07) | 1.41 (0.05) | 0.09(0.007) | 0.03 (0.008) |

**Supplementary Table 3.** The precipitation phase methods used to assess the accuracy of rain-snow partitioning approaches and to compute the average snowfall frequency and its standard deviation across the Northern Hemisphere. T_s_ is air temperature, T_d_ is dew point temperature, T_w_ is wet bulb temperature, RH is relative humidity, and P_s_ is surface pressure. Ranges for the spatially uniform thresholds were derived from the observations and relevant literature^1–4^. The ‘Map’ algorithm refers to the spatially optimized T_s_ threshold map presented in Results. Reg-Uni, Reg-Bi and Reg-Tri refer, respectively, to the optimized univariate, bivariate and trivariate binary logistic phase regression models detailed in Methods.

| **Method** | **Threshold (°C)** | **Incorporates** | | **Spatially variable** | **Mean success rate (%)** |
| --- | --- | --- | --- | --- | --- |
| T_s_-1.0 | -1.0 | T_s_ | | No | 69.3 |
| T_s_-0.5 | -0.5 | T_s_ | | No | 71.7 |
| T_s_0.0 | 0.0 | T_s_ | | No | 79.9 |
| T_s_0.5 | 0.5 | T_s_ | | No | 82.2 |
| T_s_1.0 | 1.0 | T_s_ | | No | 85.9 |
| T_s_1.5 | 1.5 | T_s_ | | No | 85.6 |
| T_s_2.0 | 2.0 | T_s_ | | No | 80.9 |
| T_s_2.5 | 2.5 | T_s_ | | No | 78.7 |
| T_s_3.0 | 3.0 | T_s_ | | No | 70.6 |
| T_d_0.0 | 0.0 | T_d_ | | No | 85.2 |
| T_d_0.5 | 0.5 | T_d_ | | No | 85.0 |
| T_d_1.0 | 1.0 | T_d_ | | No | 79.3 |
| T_w_0.0 | 0.0 | T_w_ | | No | 86.8 |
| T_w_0.5 | 0.5 | T_w_ | | No | 87.6 |
| T_w_1.0 | 1.0 | T_w_ | | No | 84.8 |
| Reg-Uni^*^ | NA | T_s_ | | No | 86.0 |
| Reg-Bi | NA | T_s_, RH | | Yes | 88.0 |
| Reg-Tri | NA | T_s_, RH, P_s_ | | Yes | 87.9 |
| Map^†^ | NA | T_s_ | | Yes | NA |
|  |  | |  |  |  |

^*^Method used in for the “Spatially continuous simulations of the 50% rain-snow T_s_ threshold” section

^†^Method used in for the “Sensitivity of snowfall frequency to phase partitioning method” section

**Supplementary Table 4.** Selected major river basins with average snowfall frequency standard deviations greater than or equal to 10.0%. The analysis only covers areas within the basin that have an annual average snowfall frequency greater than or equal to 10% (i.e., rain-only portions of the basins are not considered). When several smaller basins fall within a greater geographic area, the primary location name is used (e.g., Atlas Mountains, Central Iran, Caspian Drainage, California, and Aral Drainage).

| **Continent** | **River basin** | **Snowfall frequency σ (%)** |
| --- | --- | --- |
| Asia | Central Iran | 17.1 |
| Asia | Helmand | 16.7 |
| North America | Great Basin | 15.8 |
| Asia | Mekong | 14.5 |
| Asia | Indus | 14.3 |
| Asia | Salween | 14.2 |
| Asia | Yangtze | 14.0 |
| Asia | Tarim | 13.5 |
| Africa | Atlas Mountains | 12.6 |
| Asia | Brahmaputra | 11.6 |
| Asia | Caspian Drainage | 11.4 |
| North America | Colorado River | 11.3 |
| North America | California (west-side Sierra Nevada) | 11.3 |
| Asia | Aral Drainage | 10.2 |
| Asia | Tigris & Euphrates | 10.1 |

**Supplementary References**

1. Feiccabrino, J., Graff, W., Lundberg, A., Sandström, N. & Gustafsson, D. Meteorological Knowledge Useful for the Improvement of Snow Rain Separation in Surface Based Models. *Hydrology* **2,** 266–288 (2015).

2. Marks, D., Winstral, A., Reba, M., Pomeroy, J. & Kumar, M. An evaluation of methods for determining during-storm precipitation phase and the rain/snow transition elevation at the surface in a mountain basin. *Adv. Water Resour.* **55,** 98–110 (2013).

3. Anderson, E. A. Development and testing of snow pack energy balance equations. *Water Resour. Res.* **4,** 19–37 (1968).

4. Harpold, A. A. *et al.* Rain or snow: hydrologic processes, observations, prediction, and research needs. *Hydrol Earth Syst Sci* **21,** 1–22 (2017).

5. Stull, R. Wet-bulb temperature from relative humidity and air temperature. *J. Appl. Meteorol. Climatol.* **50,** 2267–2269 (2011).
